# Structural, Optical and Electrical Properties of Cu_0.6_Co_x_Zn_0.4−x_Fe_2_O_4_ (x = 0.0, 0.1, 0.2, 0.3, 0.4) Soft Ferrites

**DOI:** 10.3390/molecules26051399

**Published:** 2021-03-05

**Authors:** W. Aslam Farooq, Muhammad Sajjad Ul Hasan, Muhammad Iftikhar Khan, Ahmad Raza Ashraf, Muhammad Abdul Qayyum, Nafeesah Yaqub, Mona A. Almutairi, Muhammad Atif, Atif Hanif

**Affiliations:** 1 Department of Physics and Astronomy, College of Science, King Saud University, Riyadh 11451, Saudi Arabia; na-foo-sah@hotmail.com (N.Y.); mona_500@windowslive.com (M.A.A.); muhatif@ksu.edu.sa (M.A.); 2 Department of Physics, The University of Lahore, Lahore 53700, Pakistan; m.sajjadhasan@hotmail.com (M.S.U.H.); muhammad.iftikhar@phys.uol.edu.pk (M.I.K.); 3 Department of Chemistry, Division of Science and Technology, University of Education, Lahore 54770, Pakistan; raza_qau@yahoo.com (A.R.A.); hmaqayyum@ue.edu.pk (M.A.Q.); 4 Botany and Microbiology Department, College of Science, King Saud University, Riyadh 11451, Saudi Arabia; ahchaudhry@ksu.edu.sa

**Keywords:** ferrites, Co, Zn, electrical properties

## Abstract

A series of cobalt-inserted copper zinc ferrites, Cu_0.6_Co_x_Zn_0.4−x_Fe_2_O_4_ (x = 0.0, 0.1, 0.2, 0.3, 0.4) having cubic spinel structure were prepared by the coprecipitation method. Various characterization techniques, including XRD, FTIR, UV-vis and I–V were used to investigate structural optical and electrical properties, respectively. The lattice constant was observed to be decreased as smaller ionic radii Co^2+^ (0.74 Å) replaced the higher ionic radii Zn^2+^ (0.82 Å). The presence of tetrahedral and octahedral bands was confirmed by FTIR spectra. Optical bandgap energy was determined in the range of 4.44–2.05 eV for x = 0.0 to 0.4 nanoferrites, respectively. DC electrical resistivity was measured and showed an increasing trend (5.42 × 10^8^ to 6.48 × 10^8^ Ω·cm) with the addition of cobalt contents as cobalt is more conductive than zinc. The range of DC electrical resistivity (10^8^ ohm-cm) makes these nanomaterials potential candidates for telecommunication devices.

## 1. Introduction

Ferrites, a group of familiar magnetic materials with general formula MFe_2_O_4_, are used extensively in electrical, electronics, medical and, industrial applications due to their chemical constancy, economical production cost and, improved electric, magnetic and thermal characteristics. Soft ferrites are attractive materials with modest magnetization, larger anisotropy and fine electrical insulation. Many researchers have explained the optical, electrical and magnetic properties of zinc doped ferrites. Ferrites behave like insulators at room temperature and are semiconductors at a higher temperature. The electrical and magnetic properties of zinc ferrites can be improved through partial insertion of divalent ions like Cu, Co, Ni, Mn and Mg. Santosh Bhukalet et al. reported the copper-doped CoZn ferrites prepared by sol–gel auto combustion technique and found that all nanomaterials have semiconducting nature [1]. K. Anu et al. synthesized Zn-doped cobalt ferrites by applying a two-step process and investigated the variations in electrical and magnetic properties [2]. B. B. V. S. Vera Prasad et al. reported the fabrication of Cu-inserted copper zinc ferrites by auto combustion method [3]. They determined the decrease in saturation magnetization with the addition of copper contents. Muhammad Farooq Warsi et al. fabricated erbium-doped NiCo spinel ferrites by using a coprecipitation route and found adecrease in DC electrical resistivity in the range of 6.20 × 10^7^–0.03 × 10^7^ ohm-cm [4]. They observed that these materials are potential candidates for various technological devices. Sandeep B. Somvanshi et al. designed magnesium-inserted soft zinc ferrites and resolved thermal, structural, optical and spectral characteristics [5]. They concluded the increase in optical bandgap energy in the range of 1.96–2.39 eV. Structural, optical and electrical properties of La^3+^-inserted ZnMgNi soft ferrites were discussed by M. S. Hasan et al. [6].

The materials inserted in the current composition enhance the electrical and optical properties. Also, the DC electrical resistivity in the range of 10^8^ ohm-cm makes Cu-Co-Zn ferrites potential candidates for telecommunication devices.The synthesis route, size of particles and distribution of metal ions on tetrahedral and octahedral sites play vital role in determining the characteristics of soft ferrites. Zinc occupies a tetrahedral site, while the rest of metals, copper and cobalt, partially occupy tetrahedral and octahedral sites. A careful study of the literature shows that various methods have been used by researchers to synthesize the nanomaterials, such as coprecipitation, sol–gel, hydrothermal, microemulsion, mechanical milling etc. [7,8]. Coprecipitation is a popular, emerging, and economical synthesis method having enormous potential [6]. Limpidness and homogeneity are the significant properties of materials synthesized by coprecipitation that provides better impacts to this technique. In recent research work we have fabricated Co^2+^-inserted Cu_0.6_Co_x_Zn_0.4−x_Fe_2_O_4_ soft ferrites where x = 0.0, 0.1, 0.2, 0.3, 0.4 applying coprecipitationtechnique. Furthermore, structural, optical and electrical parameters of Co-inserted Cu-Zn-Ni soft ferrites were examined.

## 2. Results and Discussion

### 2.1. XRD Analysis

**Figure 1:**
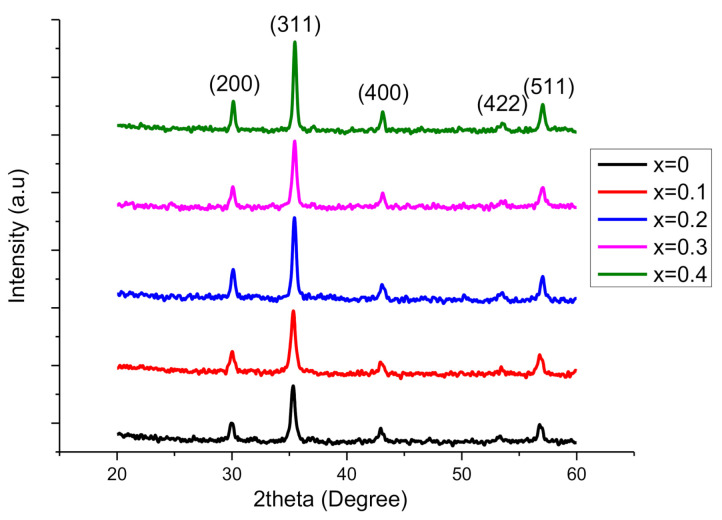
XRD patterns of Cu_0.6_Co_x_Zn_0.4−x_Fe_2_O_4_ soft ferrites where x = 0.0, 0.1, 0.2, 0.3, 0.4.

**Figure 2:**
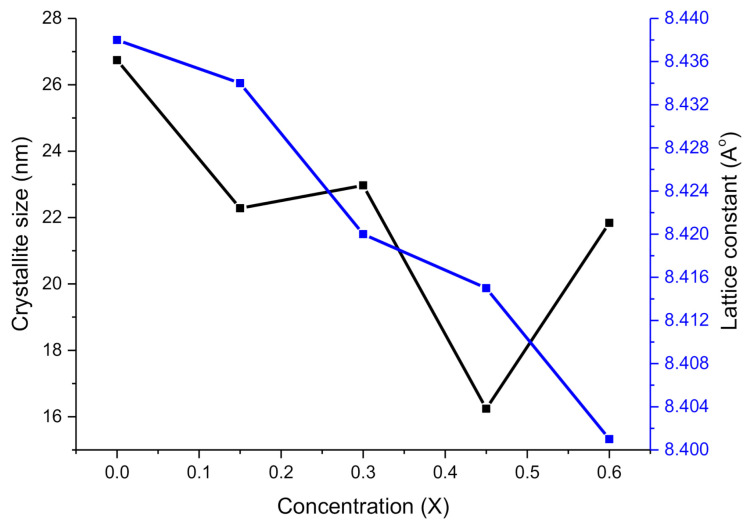
Trends for crystallite size and lattice constant with the increase of Co^2+^ concentration.

**Figure 3:**
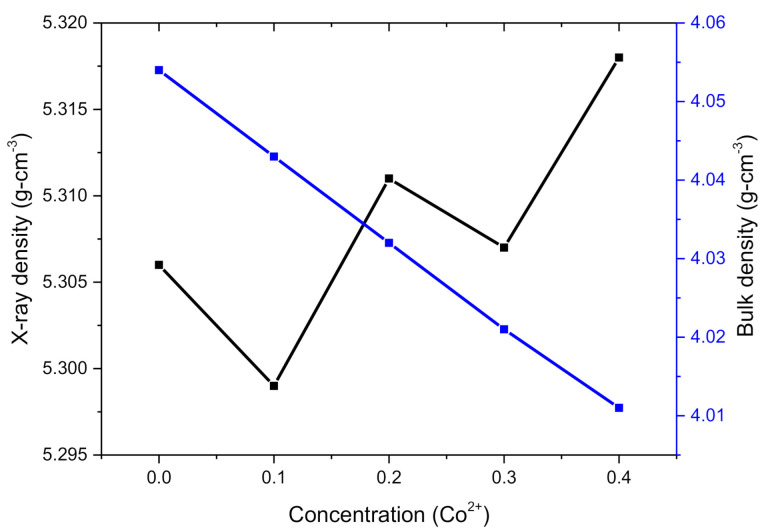
X-ray density and bulk density for Co^2+^-inserted soft ferrites.

The XRD patterns of Co-doped Cu_0.6_Co_x_Zn_0.4−x_Fe_2_O_4_ soft ferrites where x = 0.0, 0.1, 0.2, 0.3, 0.4 synthesized by coprecipitation technique are shown in Figure 1. The formation of single-phase cubic spinel structure is confirmed by prominent diffraction peaks (220), (311), (400), (422) and (511). XRD pattern confirmed that cobalt is effectively inserted into the spinel structure. By using Scherrer’s relation, the average crystallite size (*D*) was determined as [9]:

(1) $$D=\frac{0.9\lambda }{\beta cos\theta }$$

where *λ* and *β* indicate wavelength (1.542 Å) and full width at half maximum (FWHM) of peaks, respectively. Bragg’s law was used to investigate inter planer spacing or distance between crystal planes (*d*) as [10],

(2) $$d=\;\frac{n\lambda }{2sin\theta }$$

where *n* = 1 is the order of reflection for cubic structure and *θ* is the diffraction angle obtained from the XRD pattern. Table 1 shows the calculated values of average crystallite size and the lattice constant by using XRD data. Crystallite size confirmed that nanoferrites have a cubic spinel structure. The average crystallite size was determined in the range of 26.74–16.24 nm for x = 0.0 to 0.4 nanoferrites, correspondingly. Such decreasing behavior of crystallite size is due to the difference in ionic radii of Co^2+^ (0.74 Å) and Zn^2+^ (0.82 Å). Due to such difference in ionic radii during the replacement of Co by Zn on the lattice strain, some of the Co ions may reside at grain boundaries and generate stress there. Such behavior may cause a reduction in the crystallite size [11]. Lattice constant (*a*) was established by applying the equation as:

(3) $$a=\;d\sqrt{h^{2}+k^{2}+l^{2}}$$

where *d* is the interplanar spacing, (*hkl*) are Miller indices also (a = b = c) and (α = β = γ = 90°). It can be observed from Figure 1 that the most prominent peak (311) is shifting towards the right as the value of 2θ is rising. Such rising factors bring diminution in interplanar spacing, as illustrated in Table 1. Lattice constants are observed in the range of 8.438–8.401 Å. It can be examined that lattice parameters have a decreasing style with the enrichment of Co^2+^ contents. This decreasing trend is attributed to the substitution of smaller ionic radii Co^2+^ (0.74 Å) with higher ionic radii Zn^2+^ (0.82 Å). In addition, the shifting of the most prominent peak (311) and decreasing behavior of interplanar spacing causes the decrease in lattice constant. Furthermore, the doping percentage (0.1%) is very small; hence very small decreasing behavior is shown by the lattice constant. Figure 2 shows the trends demonstrated by average crystallite size and lattice constant with the increase of Cobalt contents. Various other parameters like the volume of the unit cell (*V*), X-ray density (*d_x_*) and bulk density (*d_b_*) were also determined as enlisted in Table 1 by using the relations as:

(4) $$V=\;a^{3}$$

(5) $$d_{x}=\;\frac{8M}{N_{A}V}$$

(6) $$d_{b}=\;\frac{m}{\pi r^{2}h}$$

where *M*, *N_A_* and *V* are a molecular mass of compositions, Avogadro’s number is 6.0221 × 10^23^ g mol^−1^ and volume of the unit cell, respectively. In addition, *m* is mass, *r* is the radius, and *h* is the width of nanoferrites pallets. It can be observed from the Table that the volume of the unit cells for spinel ferrites has the same declining trend as the lattice constant. X-ray density was found greater than the bulk density; however, they are presenting opposite natures by means of an increase in Co^2+^ concentration. Bulk density showed a reducing trend because Co has a lower atomic weight (58.93 amu) and density (8.86 g cm^−3^) as compared to Zn with atomic weight (65.38 amu) and density (8.91 g cm^−3^) [12]. Figure 3 demonstrates the trends of *d_x_* and *d_b_* with the increase of Co^2+^ concentration. Porosity was determined by using the relation as:

(7) $$P\left(\%\right)=\left[1-\;\frac{d_{b}}{d_{x}}\right]\times 100$$

It can be seen from Table 1 that porosity increased with the increase of cobalt contents. This parameter increase is due to the lower atomic mass of cobalt (58.93 amu) than zinc (65.38 amu).

### 2.2. Fourier-Transformation Infrared Spectroscopy (FTIR)

**Figure 4:**
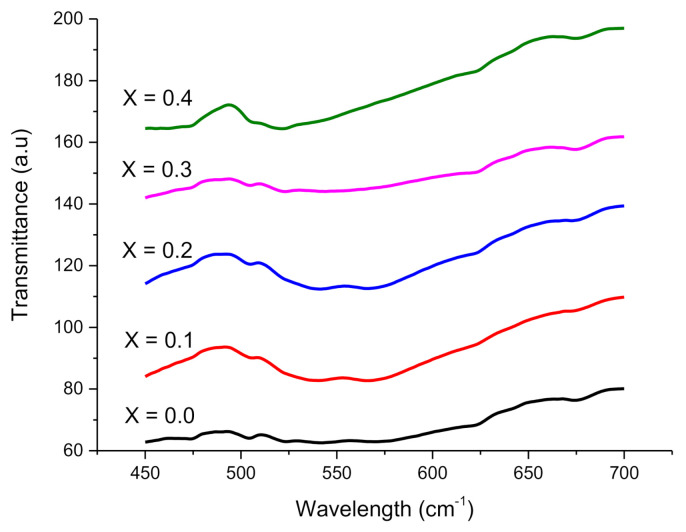
FTIR spectra of synthesized nanoferrites.

FTIR is an excellent method to examine cation allocation at tetrahedral and octahedral sites in soft ferrites. Narrow symmetries in crystalline solids and the existence or extinction of Fe^2+^ ions are also determined by this tool [13]. Two major absorption bands ν_1_ and ν_2,_ were shown by the IR spectra of the specimen as in Figure 4. Higher and lower frequency bands (ν_1_ and ν_2_) are associated with oxygen-tetrahedron (Fe-O) and oxygen-octahedron (O-Fe-O) bending vibrations, respectively [14]. Both ν_1_ and ν_2_ are mostly ascribed to Fe^3+^-(A/B)-O^2−^ vibrations due to their maximumvalency (+3) in spinel structures. The wave number ranges for ν_1_ and ν_2_ are 476.62–462.94 cm^−1^ and 540.97–524.32 cm^−1^ correspondingly with Co^2+^ insertion for present fabricated ferrites. The disparity was noticed in ν_1_ and ν_2_ with the increase in the insertion of cobalt contents. The decreasing trend shown by ν_2_ is due to altering size in the octahedron. The shifting of Fe^3+^ with Co^2+^ ions towards the octahedral site causes a decrease in ν_2_ size. In the same way, the rest of the Co^2+^ ions reside at the tetrahedral site and cause shrink in ν_1_. The alterations in Fe^3+^-O^2−^ bond length at A-site 0.189 nm and at B-site 0.199 nm are responsible for the modifications in-band locations of ν_1_ and ν_2,_ respectively [12]. The inverse spinel structure is signified by bands ν_1_ and ν_2_, where Fe^3+^ ions are dispersed at A and B sites are based on stoichiometric ratios [15].

The decrease in frequency band ν_1_ is due to the difference of ionic radii of Co^2+^ (0.74 Å) and Zn^2+^ (0.82 Å) at the tetrahedral site and M-O vibrations [12]. The locations of wave numbers (ν_1_ and ν_2_), along with the intensities and force constants, are illustrated in Table 2. The force constants were determined by the application of the following relations:

(8) $$K=4\pi^{2}\nu^{2}C^{2}m$$

where *ν*, *C* and *m* are wave number, speed of light and mass of Fe^3+^-O^2−^ ions (2.061 × 10^−23^ g), respectively. It can be observed from Table 2 that determined values of force constants for tetrahedral and octahedral bands are demonstrating decreasing trends. Such decreasing trend may be due to the decrease in ionic radii of tetrahedral and octahedral sites. In addition, the change in Fe^3+^-O^2−^ internuclear lengths alters the band positions at A and B sites [16,17].

### 2.3. UV-Vis Spectroscopy

**Figure 5:**
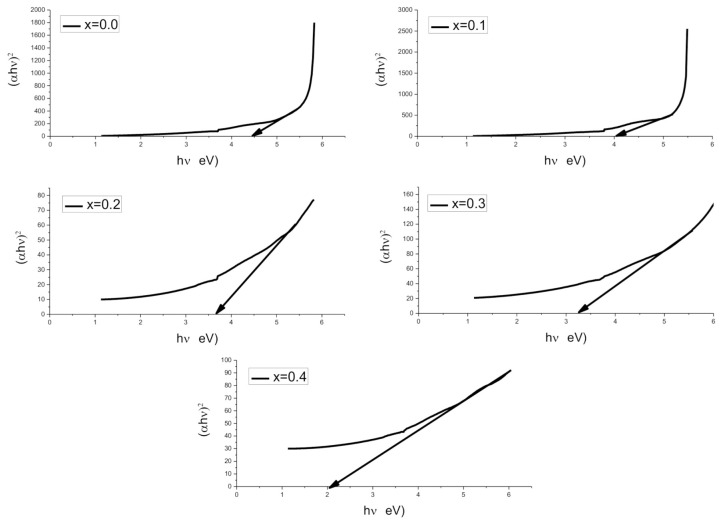
Bandgap energies (*E_g_*) of Cu_0.6_Co_x_Zn_0.4−x_Fe_2_O_4_ soft ferrites where x = 0.0, 0.1, 0.2, 0.3, 0.4.

Tauc’s relation was used to determine bandgap energy (*E_g_*) of Co^2+^-doped Cu_0.6_Co_x_Zn_0.4−x_Fe_2_O_4_ soft ferrites where x = 0.0, 0.1, 0.2, 0.3, 0.4 as given below:

(9) $$E_{g}=\frac{hv}{\lambda }$$

(10) $$\alpha hv=B{\left(hv-E_{g}\right)}^{m}$$

where *h*, *ν*, *B* and *m* are Planck’s constant, frequency and constants, respectively. In this work, *E_g_* has been obtained by drawing a plot between (*αhν*)^2^ and incident photon energy (*hν*). *E_g_* is decreased by the replacement of Zn^2+^ with Co^2+^ concentration, as represented in Tauc’s plot of Figure 5. It is due to the fact that Co is more conductive than Zn. The declining trend of *E_g_* for X = 0.00–0.60 is shown in Table 3. Furthermore, it can be observed from the figure that by the enhancement of Co contents, the curve is becoming more linear.

### 2.4. Electrical Properties

#### 2.4.1. DC Electrical Resistivity

In Co^2+^ substituted Cu_0.6_Co_x_Zn_0.4-x_Fe_2_O_4_ soft ferrites where x = 0.0, 0.1, 0.2, 0.3, 0.4 synthesized by coprecipitation technique. DC resistivity (*ρ_DC_*) was carried out by application of four-probe methods at 313 K temperature. *ρ_DC_* was investigated by employing the equation below as:

(11) $$\rho_{DC}=\;\frac{\pi }{ln2}\cdot \left(\frac{V}{I}\right)\cdot t$$

where *V* and *I* are current, and voltage and *t* is the thickness of nanoferrite pallets. Table 3 shows inspected values of DC electrical resistivities forCo^2+^-doped nanoferrites. The DC electrical resistivity (*ρ_DC_*) of Co^2+^ is 5.6 × 10^−8^ ohm-cm, while the resistivity (*ρ_DC_*) of Zn^2+^ is 5.5 × 10^−8^ ohm-cm. It can be observed that *ρ_DC_* of cobalt is greater than the zinc. Hence, the overall behavior of resistivity must be increased. It can be examined that *ρ_DC_* is increasing with the increase of Co^2+^ contents for x = 0.0 to 0.4. The hopping of electrons is responsible for the conduction process in nanoferrites. This conduction process occurs due to the hopping of electrons among Fe^2+^ and Fe^3+^ electrons. As Fe^2+^ ions partially occupy both tetrahedral A-site and octahedral B-site whereas, Co^2+^ and Zn^2+^ also partially occupy A-site and B-site. Hence, at A-site, with the increase in Co^2+^ contents, the Zn^2+^ contents at B-site decline. Hence, the movement of iron ions from A-site to B-site fulfills the lack of Zn^2+^ ions at B-site. The decrease in conduction mechanism is because of enhancement in divalent and trivalent iron ions at B-site in soft ferrites, causing the decrease in DC electrical resistivity. Hence, the decrease in DC resistivity confirms the semiconducting behavior of fabricated nanomaterials [18].

The demonstrated behavior of *ρ_DC_* is also because of factors like grain size and grain boundaries. The grains are superior conductive to the grain boundaries. The incessant series of ions makes the mobility of charge carriers easier. In addition, the resistivity has an inverse relation with the square of grain size. The reduction in grain size may increase the grain boundaries and increase the resistivity [3,19].

#### 2.4.2. Effect of Co on DC Resistivity

**Figure 6:**
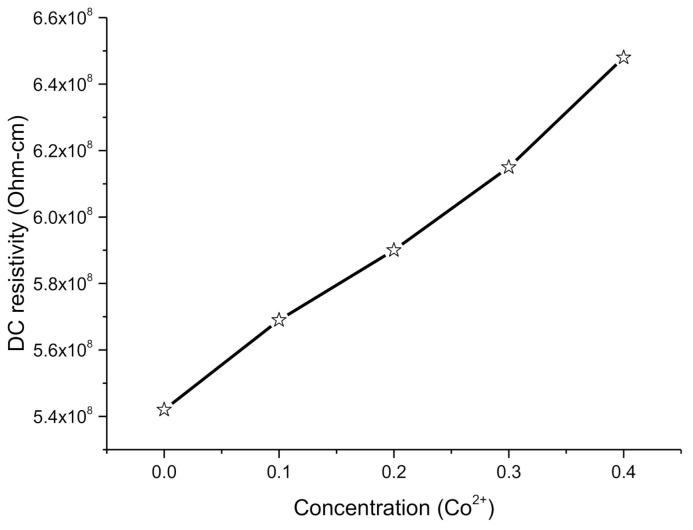
DC electrical resistivity for Cu_0.6_Co_x_Zn_0.4-x_Fe_2_O_4_ soft ferrites where x = 0.0, 0.1, 0.2, 0.3, 0.4.

The increase of Co concentration in the Co-Cu-Zn ferrites from 0.0–0.4 the DC resistivity (*ρ_DC_*) was found to increase in the range of 5.42 × 10^8^–6.48 × 10^8^ Ω·cm, as illustrated in the Arrhenius plot of Figure 6. The observed behavior of *ρ_DC_* with the increase of Co concentration can be described by Verwey and De Boer’s hopping principle [17]. This principle states that the hopping of electrons among the ions of similar elements in different valence conditions, e.g., Fe^2+^ and Fe^3+^ ions, scattered erratically over crystallographic lattice sites and create electronic conduction in ferrites [20]. The distances among the ions due to hopping and activation energy are two factors involved in the probability of hopping. The exchange interactions Co^2+^↔ Co^1+^ + e^1−^, Cu^2+^↔ Cu^1+^ + e^1−^, Zn^2+^↔ Zn^1+^ + e^1−^, Fe^3+^↔ Fe^2+^ + e^1−^, etc. are caused for p-type charge transporters in ferrite phases. Co is partially distributed on A and B sites and is responsible for partially replacing the Fe^3+^ ions on the B-site. Hence, the increasein Co ions replacement at B-site causes the decrease in Fe^3+^ ions on theB-site. Furthermore, the decrease in activation energy with the increase of Co concentration causes the few Fe ions to relocate from A to B site and reducing the Fe ions at B-site. Hence the cations switching level among Fe^2+^ and Fe^3+^ enhances. As a result, resistivity increases with the increase of Co contents inCo-Cu-Zn nanoferrites. In addition, the illustrated resistivity range (10^8^ Ω-cm) of fabricated ferrites is highly applicable in telecommunication devices [21,22].

#### 2.4.3. Drift Mobility

**Figure 7:**
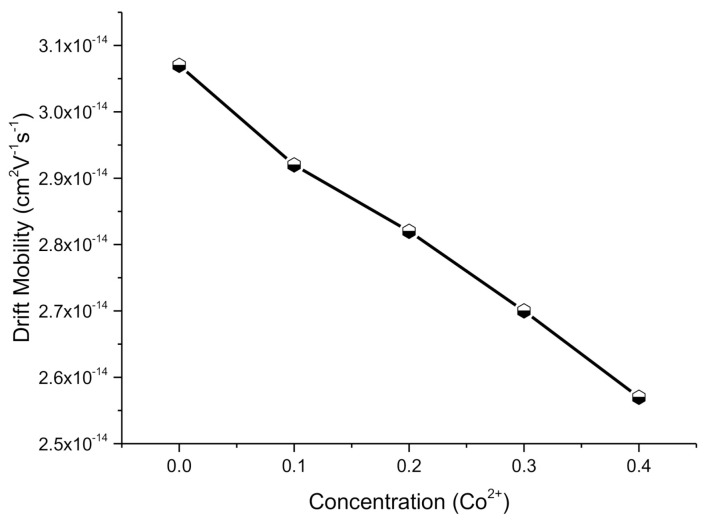
Drift mobility for Cu_0.6_Co_x_Zn_0.4−x_Fe_2_O_4_ soft ferrites where x = 0.0, 0.1, 0.2, 0.3, 0.4.

The following relation was utilized to determine the drift mobility (*μ_d_*) as:

(12) $$\mu_{d}=\;\frac{1}{\eta e\rho_{DC}}$$

where *η* and *e* are the concentration of charge carriers and charge of electrons, respectively. The concentration of charge carriers (η) can be established by using the relation as:

(13) $$\eta =\;\frac{N_{A}\rho_{b}P_{Fe}}{M}$$

where *N_A_* is the Avogadro number having value 2.022 × 10^23^ mol^−1^, *ρ_b_* is bulk density, *P_Fe_* is a number of compositional trivalent iron atoms, and *M* is the molecular weight of composed nanoferrites. The determined drift mobility (*μ_d_*) values for all Co-doped Co-Cu-Zn soft ferrites were found to increaseas enlisted in Table 3. Drift mobility reduced from 3.07 × 10^−14^ to 2.57 × 10^−14^ cm^2^V^−1^s^−1^ for x = 0.0–0.4 nanoparticles, respectively. Results indicated that the specimens with greater resistivity have short mobility and vice versa. With anincrease in temperature, the *μ_d_* decreased. The decreasing trend shown in Figure 7 shows that alteration in charge carrier mobility was due to dissimilarity in resistivity by means of temperature. Thus, with arise in temperature, the charge carriers started hopping between the sites, illustrating the increase in resistivity and enhancement in drift mobility for nanoferrites.

## 3. Materials and Methods

**Figure 8:**
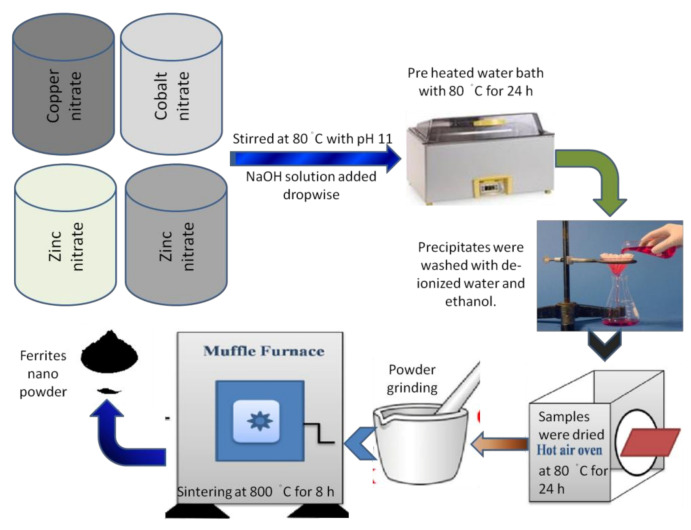
Schematic of the experimental setup.

Co^2+^-substituted Cu_0.6_Co_x_Zn_0.4−x_Fe_2_O_4_ where x = 0.0, 0.1, 0.2, 0.3, 0.4 nanoparticles were synthesized by coprecipitation method. Nitrates of copper, cobalt, zinc and iron were usedto prepare Co-doped Cu_0.6_Co_x_Zn_0.4−x_Fe_2_O_4_ nanoferrites. The stoichiometric ratios of desired salts were dissolved in deionized water. The solutions were stirred at 80 °C, and sodium hydroxide (NaOH) mixed in water was added to maintaina pH of 11. The resulting solutions were placed in a water bath at 80 °C for 24 h, followedby filtration. The particles were cleaned with deionized water followed by ethanol until apH of 7 was achieved. The obtained crystals were dried in the oven and ground into afine powder. The resulting powder of each sample was sintered at 800 °C for 8 h. The whole synthesis process is shown in Figure 8.

The structures of nanoferrites were determined using various techniques. X-ray powder diffraction (Bruker D8) scheme with Cu K_α_ supply having wavelength 1.5406 Å was used to authenticate arrangements of single-phase spinel cubic formation in all synthesized nanoferrites. Numerous structural parameters, including crystallite size, lattice constant, density (bulk and X-ray), porosityand dislocation density, were computed by XRD analysis. In order to determine the bulk density, pallets of nanoferrites were fabricated with radius (r = 0.35 cm) and width (h = 0.153 cm). For this purpose, a hydraulic press machine was used at 13 t pressure for 30 min for each pallet. UV-vis spectroscopy was used to determine bandgap energy (*E_g_*) of all specimens. Adsorption bands and force constant were calculated by (Perkin) FTIR spectroscopy. The four-probe I–V technique was utilized to study the DC electrical resistivity and drift mobility.

## 4. Conclusions

Ferrite nanoparticles were prepared by the coprecipitation method. The insertion of cobalt in Cu_0.6_Co_x_Zn_0.4−x_Fe_2_O_4_ ferrites brought novel modifications in structural, optical and electrical characteristics. XRD, FTIR, UV-vis and four-probe I–V techniques were used to characterize the samples. The lattice constant decreased with the increase of cobalt due to the smaller ionic radius of cobalt than the zinc. Crystallite size, X-ray density, bulk density and porosity were also measured. Both absorption bands (tetrahedral and octahedral), along with their corresponding intensities, decreased with the increase of cobalt contents leading to adecrease in force constants for composed nanoferrites. DC electrical resistivity increased with the increase of cobalt concentration, confirming the semiconductor nature of composed nanoferrites. The determined range of DC electrical resistivity indicated that these fabricated materials are highly applicable in telecommunication devices.

## Figures and Tables

**Table 1:** *2θ* of (311) peak, lattice constant (*a_exp_*), crystallite size (*D*), unit cell volume (*V*), d-spacing (*d*), X-ray and bulk densities (*d_x_* and *d_b_*) and porosity (*P*) for Cu_0.6_Co_x_Zn_0.4−x_Fe_2_O_4_ soft ferrites where x = 0.0, 0.1, 0.2, 0.3, 0.4.

Parameter	X = 0.00	0.1	0.2	0.3	0.4
*2θ* of (311) peak	35.32	35.34	35.40	35.43	35.48
*a_exp_* (Å)	8.438	8.434	8.420	8.415	8.401
*D* (nm)	26.74	22.28	22.97	16.24	21.84
*V* (Å)^3^	600.78	599.93	596.98	595.88	592.91
*d* (Å)	2.544	2.542	2.538	2.535	2.533
*d_x_*(g-cm^−3^)	5.306	5.299	5.311	5.307	5.318
*d_b_* (g-cm^−3^)	4.054	4.043	4.032	4.021	4.011
*P* (%)	23.59	23.70	24.08	24.23	24.57

**Table 2:** FTIR band spectrum showing absorption bands (ν_1_ and ν_2_), intensities (I_1_ and I_2_) and force constants (K_T_ and K_o_).

X	V_1_ (cm^−1^)	I_1_ (%)	K_T_ × 10^5^ (Dyne cm^−1^)	V_2_ (cm^−1^)	I_2_ (%)	K_o_ × 10^5^ (Dyne cm^−1^)
0.0	540.97	62.74	2.15	476.62	64.05	1.66
0.1	538.73	59.73	2.13	473.83	67.19	1.64
0.2	537.49	54.44	2.12	472.86	62.11	1.63
0.3	524.77	62.31	2.03	467.87	63.14	1.60
0.4	524.32	47.46	2.02	462.94	47.27	1.56

**Table 3:** Bandgap energy (*E_g_*), electrical resistivity (*ρ_DC_*) and drift mobility (*µ_d_*) for nanoferrites.

Parameters	Results				
X	0.0	0.1	0.2	0.3	0.4
*E_g_* (eV)	4.44	4.05	3.64	3.23	2.05
*ρ_DC_*(Ω·cm)	5.42 × 10^8^	5.69 × 10^8^	5.90 × 10^8^	6.15 × 10^8^	6.48 × 10^8^
*µ_d_* (cm^2^V^−1^s^−1^)	3.07 × 10^−14^	2.92 × 10^−14^	2.82 × 10^−14^	2.70 × 10^−14^	2.57 × 10^−14^

## Data Availability

No new data were created or analyzed in this study.

## Sample Availability

Not available.
